# Feature Engineering and a Proposed Decision-Support System for Systematic Reviewers of Medical Evidence

**DOI:** 10.1371/journal.pone.0086277

**Published:** 2014-01-27

**Authors:** Tanja Bekhuis, Eugene Tseytlin, Kevin J. Mitchell, Dina Demner-Fushman

**Affiliations:** 1 Department of Biomedical Informatics, School of Medicine, University of Pittsburgh, Pittsburgh, Pennsylvania, United States of America; 2 Lister Hill National Center for Biomedical Communications, National Library of Medicine, U.S. National Institutes of Health, Bethesda, Maryland, United States of America; University Hospitals of Geneva, Switzerland

## Abstract

**Objectives:**

Evidence-based medicine depends on the timely synthesis of research findings. An important source of synthesized evidence resides in systematic reviews. However, a bottleneck in review production involves dual screening of citations with titles and abstracts to find eligible studies. For this research, we tested the effect of various kinds of textual information (features) on performance of a machine learning classifier. Based on our findings, we propose an automated system to reduce screeing burden, as well as offer quality assurance.

**Methods:**

We built a database of citations from 5 systematic reviews that varied with respect to domain, topic, and sponsor. Consensus judgments regarding eligibility were inferred from published reports. We extracted 5 feature sets from citations: alphabetic, alphanumeric^+^, indexing, features mapped to concepts in systematic reviews, and topic models. To simulate a two-person team, we divided the data into random halves. We optimized the parameters of a Bayesian classifier, then trained and tested models on alternate data halves. Overall, we conducted 50 independent tests.

**Results:**

All tests of summary performance (mean F3) surpassed the corresponding baseline, *P*<0.0001. The ranks for mean F3, precision, and classification error were statistically different across feature sets averaged over reviews; *P*-values for Friedman's test were .045, .002, and .002, respectively. Differences in ranks for mean recall were not statistically significant. Alphanumeric^+^ features were associated with best performance; mean reduction in screening burden for this feature type ranged from 88% to 98% for the second pass through citations and from 38% to 48% overall.

**Conclusions:**

A computer-assisted, decision support system based on our methods could substantially reduce the burden of screening citations for systematic review teams and solo reviewers. Additionally, such a system could deliver quality assurance both by confirming concordant decisions and by naming studies associated with discordant decisions for further consideration.

## Introduction

Comparative effectiveness research (CER) identifies the best treatments, devices, diagnostic tests, and policies for patient care. Various stakeholders use the information garnered in CER to guide their healthcare decisions. Thus, timely CER of high quality is essential for evidence-based medicine (EBM) [1]. In addition to primary research, much of EBM rests on secondary research, such as synthesis of medical evidence [2]. To date, an important source of synthesized evidence resides in the global corpus of systematic reviews (SRs), mainly supported by large organizations such as the Cochrane Collaboration [3] and the US Agency for Healthcare Research and Quality [4]. Although traditional reviews are preponderant [5], growth in production of SRs is accelerating. For example, the Cochrane Database of Systematic Reviews includes 5,591 SRs, an almost six-fold increase since the year 2000 [6]. In 2010, Bastian et al [5] reported that while 75 trials and 11 SRs are published daily, synthesis seriously lags report of evidence in trials. Clearly, a significant challenge in translational research is synthesizing scientific output in a timely manner.

A major bottleneck in producing SRs involves labor-intensive, dual screening of citations and articles [7]. Screening entails two phases where, in a best-case scenario, at least two reviewers independently screen the entire set of citations to identify *provisionally* eligible studies. Then, at least two reviewers read the full-texts of reports to determine which studies to include in their review. The reason for dual review in each phase is to control human error and bias in judgments. Both the Institute of Medicine (IOM) and the Patient-Centered Outcomes Research Institute (PCORI) see quality assurance as desirable. However, recognizing that screening twice may be infeasible, PCORI softened the IOM requirement of dual review (IOM standard 3.3.3) [8], stating “fact-checking may be sufficient” (PCORI SR-1 standard, p. 5) [9].

The long-term goal of our programmatic research is to support systematic reviewers by reducing the burden associated with screening citations and by offering guidance as to which studies to reconsider based on their judgments, effectively offering quality assurance or ‘fact-checking.’ Our past and current research is novel in that we model the first screening phase and use as our gold standard judgments based on screening citations rather than judgments based on reading full-text reports. We conduct independent experiments over several kinds of reviews to demonstrate generalizability. Moreover, we focus on reviews where nonrandomized or observational studies are eligible for inclusion because the screening burden is likely to be greater than for reviews restricted to randomized controlled trials [10], [11].

A handful of research groups are working on closely related problems, such as reducing the labor associated with regularly updating reviews, prioritizing work when creating and maintaining SRs, and improving a Bayesian algorithm for imbalanced data [12]–[15]. Perhaps, the most closely related research is by Cohen et al [16], Wallace et al [17], and Frunza et al [18]. However, these studies differ from ours with respect to the nature of the gold standard, study design, performance measures, or vision for a support system. Bekhuis and Demner-Fushman [10] present a more detailed review of research on automating screening methods. An interesting plan for a text-mining pipeline to support SR production is described by Cohen et al [19] with modules to reduce screening burden.

**Figure 1:**
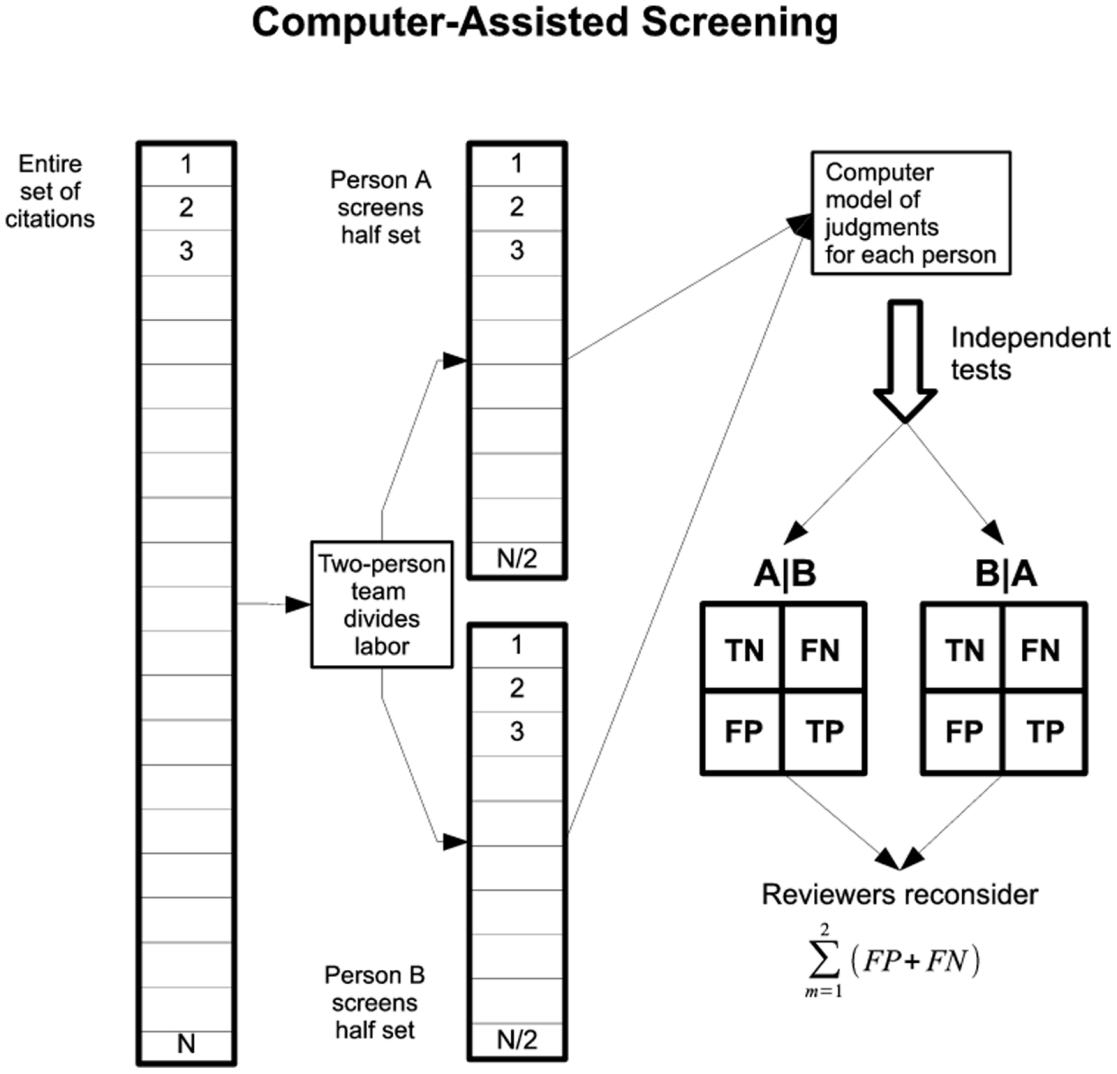
Computer-assisted screening task. Depicts a computer-assisted, decision support system for systematic reviewers. Instead of screening an entire set of citations twice, reviewers divide the labor. The system could further reduce screening burden, as well as offer quality assurance by confirming concordant decisions and naming studies that need to be reconsidered. A and B are random halves of the citations from a review. A|B = independent test of classifier on A dataset given model from training on B; B|A = independent test of classifier on B dataset given model from training on A. TN = true negative; FN = false negative; TP = true positive; FP = false positive; m = confusion matrix that displays classification results for an independent test.

Our earlier efforts to classify studies with respect to eligibility for inclusion in SRs rested on bits of alphabetic text appearing in full citations (titles, abstracts, and metadata) [10]. For the work described here, we developed various kinds of feature sets capitalizing on alphanumeric and semantic information, latent structure in citations, and concepts in blocks of full-text from SRs. We then trained and tested a classifier suitable for imbalanced data, compared performance over feature sets, and demonstrated that screening burden can be substantially reduced. We also describe a computer-assisted decision support system and future development based on performance results and analysis of classification errors (Figure 1).

## Methods

### Database

Our nonprobability sample consists of 5 SRs where reviewers reported that nonrandomized or observational studies were eligible for inclusion. Further, reviewers had to have screened at least 1000 citations and identified at least 1% as provisionally eligible for full-text review. The latter criterion was essential as a large number of reviews are empty or infeasible, i.e., investigators find zero or 1 eligible study [20], [21]. The reviews cover various domains, topics, and sponsoring organizations, including the Cochrane Collaboration and the US Agency for Healthcare Research and Quality (AHRQ). Two are diagnostic (detection of malaria [22] and galactomannan for invasive aspergillosis [23]); two are therapeutic (treatment of ameloblastoma of the jaws [24] and monitoring the effect of an antibiotic in patients with organ transplants [25]); and one is epidemiological (prevention of influenza in the elderly [26]).

To build a database of citations, we re-ran MEDLINE [27] and Embase [28] searches that appeared in the reviews, limiting to records added no later than the reported search dates. This limit precluded retrieving citations for studies that could have been eligible, but were not seen by the reviewers. We also enriched retrieval sets by manually searching for provisionally included studies that were not automatically retrieved. This was important because the percentage of eligible studies is typically relatively small and eligible studies are the positive examples we wished to identify. Note that MEDLINE and Embase searches returned citations with titles, abstracts, and metadata rather than full texts, which mirrored the experience of the review teams. Additionally, we retrieved full texts for the reports in which the searches appeared. The database therefore consists of published, full-text reports for 4 SRs and 1 protocol, and datasets for citations that would have been retrieved by the reviewers. On average, we recovered 94% of the citations screened by the review teams.

We labeled citations as *include* or *exclude* based on published flow charts, tables, and reference lists. Thus, the labels reflect the consensus judgments of reviewers, each of whom has domain expertise.

### Feature sets

Given our earlier findings [10], we overweighted titles by writing the title twice in each full citation.

We then extracted the following five feature sets per review:

#### Alphabetic features

We converted text to lower case, tokenized on non-alphabetic characters and white space, deleted stop words, selected tokens (features or strings of text) between 3 and 100 characters long, and Porter stemmed to normalize [29]. Porter stemming strips suffixes so that morphological variants of words map to the same stem or root. For example, *vaccination* and *vaccines* map to *vaccin* after stemming. We pruned tokens occurring in fewer than 3 citations. Most of the features were unigrams (single tokens). However, we also extracted bigrams (adjacent pairs of tokens) from titles to further overweight information from this field.

#### Alphanumeric features^+^

We extracted features with embedded numbers or punctuation, such as *h3n2*, *a/fujian/411/2002*, and *case-control*. We also extracted alphabetic features without stemming, journal names, bigrams from titles, and strings of numbers interrupted by punctuation, such as *2004–2005*. We replaced em and en dashes with hyphens and did not prune.

#### Indexing terms

We extracted terms in the indexing field by matching regular expressions (see Software section). Terms came from MeSH [30] or Emtree [31], the controlled vocabularies for MEDLINE and Embase, respectively.

#### Concepts in SRs

This feature set consists of concepts that review authors used to describe their research topic and criteria for inclusion and exclusion of studies. For example, we identified concepts for patients, conditions, treatments, diagnostic tests, outcomes of interest, and study designs. This strategy broadly followed the well-known Patient, Problem, Intervention, Comparison, and Outcome (PICO) strategy [32], [33] used by researchers to structure their comparative effectiveness questions. We used titles and blocks of text from the abstracts and methods sections. We also used snippets of text in the introductions to disambiguate acronyms. To build a lexicon of concepts appearing in the excerpts, we used the Metathesaurus of the NLM Unified Medical Language System v. 2012AB [34] in combination with an in-house version of IndexFinder [35]. We enriched the SR lexicon with direct semantic neighbors of concepts, as well as study designs from our terminology [36], [37]. The latter was necessary as MeSH exactly covers just 19% of the terms that methodologists use for study designs and most of the missing terms do not appear in any other UMLS resource [36]. We also used our version of IndexFinder [35] to locate concepts appearing in citations, after splitting on lines.

#### Topic model features

To explore whether latent topics could be useful for classification, we fit Latent Dirichlet Allocation (LDA) topic models to citations per review using alphanumeric^+^ features. LDA, introduced by Blei, Ng, and Jordan [38], extends Probabilistic Latent Semantic Analysis [39] by placing a Dirichlet prior on the distribution of topic probabilities. For interested readers, we recommend a paper by Steyvers and Griffiths [40]. Before we could use topics (T) as features, we had to find an optimal T per review. To do this, we implemented a method described in [41] setting the Dirichlet hyperparameters alpha = 50/T and beta = 0.1, varying T. Choosing T is a model selection problem where the best T maximizes the log likelihood of the model given the data. Here, the data were the alphanumeric^+^ features or ‘words’ (w) in the corpus. We therefore needed to compute the P(w|T), which is computationally intractable. However, one can approximate P(w|T) by computing the harmonic mean over a set of samples from P(w|z,T), where z is a vector of word assignments to T. Assignments z are sampled from the posterior distribution P(z|w,T). To generate samples, we used a Gibbs sampler, a Markov chain Monte Carlo algorithm available in the Mallet Toolkit [42], and then computed the likelihoods using equation 2 in Griffiths and Steyvers, p. 5229 [41]. Once we selected T and used the settings for alpha and beta just described, we fit topic models per review and computed Kullbach-Leibler (KL) divergences using software we developed to build datasets for machine learning (see Software section).

Each citation was represented as a vector of features with the following weights: term frequency x inverse document frequency (p. 109, [43]) for the alphabetic, alphanumeric^+^, and indexing sets; term frequency for concepts in SRs; and topic probabilities plus KL divergences for the topic model set. We reduced set size by selecting features if information gain was ≥0.001.

### Software

To extract features from citations, we developed standalone Java programs or used RapidMiner v.5.2 [44], [45]. We also developed an Evidence in Documents, Discovery, and Analysis (EDDA) extension with two open source plugins written in Java for the RapidMiner (RM) community [46]. One plugin is a wrapper that integrates topic-modeling code from the MALLET Toolkit [42] into RM processes. The user can build a topic model for a corpus of text files and compute KL divergences to compare probability distributions for citations and classes; our implementation is symmetric [40]. The distributions are defined by the topic probabilities for a given citation as compared to the median topic probabilities for the *include* class or the *exclude* class. This operator produces a citation by feature dataset with topics and/or KL divergences as features. The second plugin integrates the Java Regex utility for regular expressions into RM text processes. It produces a citation by feature dataset based on a series of Regex matches, where each regular expression is a feature. The EDDA extension is freely available online [46].

### Study design

**Figure 2:**
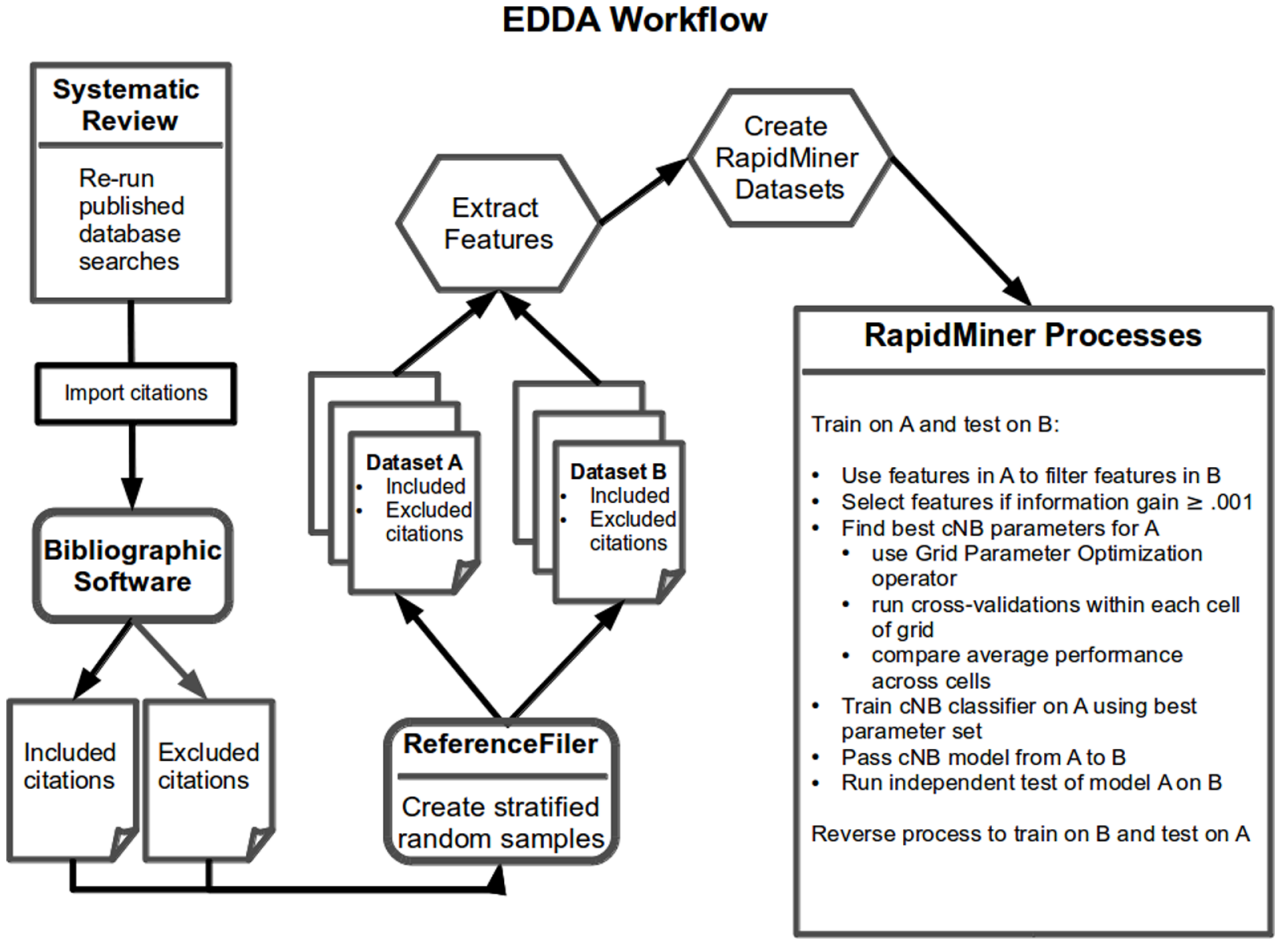
EDDA workflow. An overview of the project workflow. EDDA = Evidence in Documents, Discovery, and Analysis. Reference Filer = in-house Java program that sorts citations into folders; resultant datasets A and B are random halves of the citations stratified with respect to eligibility for provisional inclusion in a systematic review; citations include titles, abstracts, and metadata. RapidMiner is an open source, data mining suite. cNB = Weka complement naïve Bayes classifier available in Rapid Miner; suitable for imbalanced data typical of systematic reviews. Grid Parameter Optimization operator searches for best performance over a grid; dimensions based on combinations of parameter settings.

We used a Weka complement naïve Bayes (cNB) classifier [47] available in RM that is suitable for imbalanced data where one class is much smaller than the other. To find the best set of cNB parameters for normalization and smoothing, we used the RM Grid Parameter Optimization operator. This operator returned the parameter set associated with best average performance over the cells of a grid. The size of the grid was determined by all combinations of our settings for normalization (true, false) and smoothing (.001, .250, .500, .750, 1.0). Within each of 10 cells in the grid, we ran 5×2-fold cross-validations, where each fold was stratified with respect to percentage of eligible studies. Thus, the total number of iterations was 100. We trained the classifier on half the data using the best parameter set and then conducted an independent test on the other half. Note that we used ReferenceFiler, an in-house Java program, to randomly select citations in a retrieval set to split the data in half, stratifying with respect to the percentage of studies eligible for provisional inclusion in a given review (Figure 2).

An important aspect of this design is that we optimized the parameters for the cNB classifier with respect to recall and precision *simultaneously*. To do this, we optimized with respect to F3, a summary measure of performance and weighted harmonic mean that overweights recall relative to precision (see [43], p. 144):
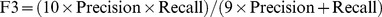
(1)Using F3 rather than recall with a floor for precision as we did in [10] substantially reduced the total number of misclassified studies, especially the number of false positives, and was still faithful to reviewer behavior when screening citations. Additionally, this design facilitated direct estimation of reduction in screening burden (RSB) and yielded stable estimates of performance because we could average outcomes over the two tests per review.

To simulate a two-person team where each person screened half the citations once, we randomly split the data into two halves (A and B). We then trained on A and tested on B and vice versa, preserving the machine-learning paradigm of independent tests. The rationale for the A|B and B|A setup is that training on half the data simulated building a model of judgments for one reviewer that could then be applied to the other reviewer's judgments. Misclassified studies therefore could reflect discordant decisions between persons A and B.

We assume by virtue of our design that RSB for the first pass through the citations was 50% because review of the entire set was conducted once instead of twice. In this study, we focused on RSB for the second pass through the citations, which we operationalized as simply (100% – *mean* classification error %). Mean classification error equals (100% – accuracy %) averaged over the independent tests. More specifically, it equals the percentage of (false positives+false negatives) averaged over the A|B and B|A tests.

Overviews of the computer-assisted screening task and study workflow appear in Figures 1 and 2, respectively.

### Evaluation of performance

Reviewers are overly inclusive when they screen citations because they are loath to exclude a study prematurely. In other words, they maximize recall at the expense of precision during the first screening phase. Consider, if we trained a classifier to label all studies as *include*, recall would be perfect, precision would be the percentage of provisionally eligible studies, and all the errors would be false positives. However, the cost of perfect recall implies maximal screening burden and uninformative feedback—‘uninformative’ because no feedback is possible regarding excluded studies, both true and false negatives. To be worthwhile, a classifier must return performance better than this baseline to ensure reduced labor and informative feedback.

We conducted 50 independent tests: 5 reviews ×5 feature sets ×2 tests (A|B and B|A). We statistically compared overall mean performance (F3) for cNB by feature set and review to a baseline F3 (assuming recall = 100% and precision = % eligible). To compare performance across feature sets, we computed nonparametric tests of mean recall, precision, F3, and classification error using Friedman's two-way analysis of ranks for related samples where the two ‘ways’ were reviews and feature sets [48].

## Results

### EDDA database

The median total number of citations retrieved across datasets was 2846 (range: 1816 to 10796). The median percentage of provisionally eligible studies was 5.8% (range: 4.3% to 12.5%). The percentage of included studies was similarly distributed across the A and B random halves. (Table 1)

**Table 1:** Number and allocation of citations per systematic review.

	Influenza	Malaria	Galacto^a^	Organ Trans^b^	Ameloblastoma
A exclude	2593	1245	1052	5155	811
A include	154	177	47	243	57
Subtotal (% eligible)^c^	2747 (5.6%)	1424 (12.4%)	1100 (4.3%)	5398 (4.5%)	868 (6.7%)
B exclude	2575	1246	1053	5154	890
B include	163	178	47	244	58
Subtotal (% eligible)	2738 (6.0%)	1422 (12.5%)	1099 (4.3%)	5398 (4.5%)	948 (6.1%)
Total (% eligible)	5485 (5.8%)	2846 (12.5%)	2199 (4.3%)	10796 (4.5%)	1816 (6.3%)

a Galacto = Galactomannan.

b Organ Trans = Organ Transplant.

c % eligible = percentage provisionally eligible for inclusion in a review; judgments based on screening citations (titles and abstracts) by domain experts.

### Feature sets

The alphanumeric^+^ feature set was much larger than the other sets. (Table 2) However, size was considerably reduced after filtering by information gain. This was primarily because many of the alphanumeric^+^ features were uninformative strings of numbers with internal punctuation. The SR concepts set was the smallest followed by the topic model set. Note that fitting topic models substantially reduced the dimensionality of the alphanumeric feature space. A sample of features by type for the influenza review is displayed in Table 3.

**Table 2:** Feature set size by systematic review before and after filtering for information gain.

N features (n if IG≥0.001)^a^					
	Alphabetic	Alphanumeric^+^	Indexing	Topic model	SR concepts^b^
**Influenza**					
A^c^	6880 (4759)	52013 (10404)	5392 (5251)	1602 (1601)	821 (681)
B	6982 (4740)	52231 (13043)	5361 (5226)	1602 (1601)	821 (697)
**Malaria**					
A	4901 (3274)	35947 (10481)	1391 (1353)	902 (901)	575 (519)
B	4937 (3249)	36375 (10272)	1382 (1318)	902 (901)	575 (531)
**Galactomannan**					
A	4026 (2757)	27960 (6423)	1012 (1000)	602 (594)	449 (359)
B	4057 (2761)	27561 (6422)	1060 (1048)	602 (601)	449 (352)
**Organ Transplant**					
A	11765 6680)	72807 (15005)	5577 (5435)	1902 (1894)	793 (571)
B	11856 6294)	72529 (13872)	5521 (4617)	1902 (1901)	793 (587)
**Ameloblastoma**					
A	3247 (2303)	19311 (5043)	563 (556)	602 (601)	351 (279)
B	3352 (2422)	20712 (5311)	611 (601)	602 (600)	351 (290)

a IG = information gain.

b SR = systematic review.

c A and B refer to random halves of the data.

**Table 3:** Sample of features by type for the influenza review.

Alphabetic	Alphanumeric^+^	Indexing	Topic model	SR concepts
ag	aged	*Aged	**topic_00001**	Old_age
elderli	elderly	Aged	cells	Elderly_population_group
influenza	influenza	*Influenza Vaccines	autologous	Influenza_vaccination
vaccin	vaccines	Influenza Vaccines	virology	Aged_80_and_over
influenza_vaccin	influenza_vaccination	elderly	measured	prevention_control
epidemiolog	epidemiology/prevention	80 and over	virus-specific	Nursing_Homes
agent	vaccines/adverse	immunology	t-lymphocytes	therapeutic_aspects
advers	agents/ae	Influenza A virus/	cytotoxicity	Vaccines
epidem	h3n2_epidemic	(Antigens, Viral)	activity	Sudden_death
Case	case-control	Serology and Transplantation	ctl	Mortality_Vital_Statistics
Control	1990–1991	Case-Control Studies	cytotoxic	Respiratory_Tract_Infections
Commun	community-dwelling	case report	…	historical_cohort_design
Sydnei	a/Sydney/05/97	adverse effects	**topic_00002**	case_comparison_design
Journal	new_england_journal_of_medicine	147205-72-9 (CD40 Ligand)	observed	Chronic_obstructive_asthma_with …
Blind	double_blinded	Interferon-gamma/bi	cytokines/bl	
			cytokine	
			il-10	
			obtained	
			il-6	
			assay	
			results	
			blood	
			cytokines	
			…	
			**topic_01600**	
			…	
			include_divergence	
			exclude_divergence	

**Note:** For alphabetic and alphanumeric^+^ sets, features with an underscore between pairs of words came from titles. For the indexing set, features mainly came from MeSH and Emtree; an asterisk indicates a major concept. For topic model set, number of topics determined prior to training (see Methods); based on alphanumeric^+^ features; Kullbach-Leibler (KL) divergences from mediods for include or exclude class. SR = systematic review. For SR concepts, lexicon consists of UMLS concepts (including parent and children) in SRs and study design terms.

### Performance

All tests of summary performance (mean F3) surpassed the corresponding baseline F3: one-tailed *Z*-tests, *Z* ranged from 24.35 (ameloblastoma, SR concepts) to 72.06 (organ transplant, alphabetic), *P*<0.0001. Baseline F3 values appear online in a supplementary table (Results S1). Over all conditions, mean F3 ranged from 52.52% to 90.73%; mean recall from 59.68% to 96.81%; mean precision from 13.82% to 72.38%; and mean classification error from 1.91% to 26.08%. (Table 4) Mean RSB for the second pass through the citations ranged from 73.92% to 98.09% (Results S1).

**Table 4:** Mean performance of the cNB classifier by systematic review and feature set.

	Alphanumeric^+^	Alphabetic	Topics	SR concepts	Indexing
**F3 (%)** a					
Ameloblastoma	75.11	74.52	71.51	68.22	68.68
Influenza	65.52	57.16	61.97	59.38	63.11
Galactomannan	87.31	90.73	74.73	78.88	74.13
Malaria	88.09	89.30	86.42	83.33	81.85
Organ transplant	57.82	64.39	59.17	54.24	52.52
*Mean rank* b *^, ^* d	*4.20*	*4.00*	*3.00*	*2.00*	*1.80*
**Recall (%)**					
Ameloblastoma	80.01	79.98	78.27	87.78	81.76
Influenza	76.44	59.68	73.63	76.33	77.70
Galactomannan	89.37	96.81	96.81	95.74	92.55
Malaria	90.98	95.77	93.80	92.67	90.69
Organ transplant	59.77	71.87	74.95	74.14	80.31
*Mean rank* b *^, ^* e	*2.20*	*2.90*	*3.10*	*3.40*	*3.40*
**Precision (%)**					
Ameloblastoma	49.15	46.47	40.39	26.89	28.21
Influenza	30.08	42.00	25.83	19.82	23.50
Galactomannan	72.38	58.12	24.51	30.51	26.64
Malaria	68.75	55.55	50.60	43.73	43.78
Organ transplant	46.05	33.25	20.45	17.05	13.82
*Mean rank* b *^, ^* d	*4.80*	*4.20*	*2.60*	*1.60*	*1.80*
**Classification error (%)**					
Ameloblastoma	6.66	7.15	8.73	20.31	14.39
Influenza	12.31	7.16	13.82	19.24	15.90
Galactomannan	1.91	3.14	12.92	9.51	11.33
Malaria	6.33	10.09	12.20	15.78	15.74
Organ transplant	5.06	7.78	14.29	18.90	26.08
*Mean rank* c *^, ^* d	*1.20*	*1.80*	*3.40*	*4.40*	*4.20*

a Baseline F3 (%): Ameloblastoma = 40.20; Influenza = 38.96; Galactomannan = 31.00; Malaria = 58.82; Organ transplant = 32.03. All mean F3 values surpassed the baseline values, one-tailed Z-tests, P<0.001.

b Higher ranks associated with better performance.

c Lower ranks associated with better performance.

d Mean ranks significantly different for F3, precision, and classification error: Friedman's test of mean F3 ranks (4 df) = 9.760, *P* = .045; Friedman's test of mean precision ranks (4 df) = 16.480, *P* = .002; Friedman's test of mean classification error ranks (4 df) = 16.480, *P* = .002.

e Friedman's test of mean recall ranks (4 df) = 1.980, *P* = .739, NS.

The ranks for mean F3, precision, and classification error were statistically different across 5 feature sets averaged over 5 reviews; the *P*-values for Friedman's test were .045, .002, and .002, respectively. (Table 4) Differences in ranks for mean recall were not statistically significant (*P* = .739). The following is an ordered list of feature types arranged from high to low relative to mean F3: alphanumeric>alphabetic>topics>SR concepts>indexing. Remarkably, performance associated with terms in citations assigned by indexers was very similar to performance associated with concepts extracted from full-text reports written by systematic reviewers. Note that each dataset in our database has a corresponding published report (4 SRs and 1 protocol). The selected text blocks from the SRs would have appeared in earlier protocols and, as such, include information available to the reviewers when they screened citations.

Follow-up pairwise comparisons for significant tests of ranks averaged over reviews tended to be significant when comparing smaller feature sets to larger ones; comparisons for alphabetic vs alphanumeric features and SR concepts vs indexing were always nonsignificant. However, a more nuanced analysis by feature type and review revealed quite variable performance, which suggests that two-way interactions exist (Results S1).

## Discussion

In this study, we improved earlier research [10], [49] by *not* using performance criteria better suited for information retrieval filters, e.g., see papers from the Haynes group [50], [51]. Instead, we relaxed the insistence on very high recall (sensitivity) and substantially reduced the total number of misclassified studies, both false positives and false negatives. Additionally, we assumed that humans screened a complete set of citations once rather than twice and that labor was divided between teammates. Division of labor is particularly likely for large reviews. For example, a recent study of medication management involved dual screening of about 33,000 citations by a team of reviewers (see Figure 1 in McKibbon et al [52]). Assuming a single complete review of citations with division of labor distinguishes our approach from Frunza et al [18] and from those involving active learning [17], a method that obviates the need for a complete set of labels, but requires interactive feedback and acceptance of automated exclusion. It is unclear which type of system will be adopted by systematic reviewers in the future, especially since head-to-head comparisons are not possible given differences in system design and performance measures. However, our system is in keeping with current PCORI standards, as it will offer computer-assisted decision support to resolve discordant decisions when a dual review is not feasible [9].

Interestingly, our setup for a two-person team could generalize to both larger teams and solo reviewers. For example, if more than two people were to split the screening task, we could model pairs of reviewers as we did here. If the team consisted of an odd number of people, we could apply the model from one person more than once. Identifying an expert could also be useful for training, as we could point out discordant decisions between expert and novice. If the initial list of labels were based on just one reviewer's judgments—which is not uncommon—we could still use this setup to point out where the computer disagrees with the reviewer, presumably because the person was inconsistent. Regardless of team size, discordant decisions may indicate human fallibility. This is not far-fetched given our analyses of misclassified studies (see below).

Based on the findings of this study, the envisioned production system could reduce labor by 88% to 98% for the second pass through the citations if we were to use the alphanumeric^+^ feature set (Results S1). This is markedly better than our earlier results [10]. If one considers the 50% reduction in labor by assuming a single rather than dual review, the overall reduction in labor is 38% to 48%. Reviewers would not consider further the very large set of TNs, but would discuss discordant decisions (FPs and FNs) to reach consensus. They would then have a set of provisionally positive studies consisting of the TPs returned by the system and a handful of studies identified in their reconsideration of discordant decisions. In the final phase, they would read the full texts of provisionally positive studies in the adjusted set to make a final adjudication regarding inclusion in the review.

### Feature sets

To understand how performance might depend on the type of feature, we compared five different sets with the goal of selecting the best set for future development of a production system. Although tests of ranked performance averaged over reviews suggested that the alphanumeric^+^ set was best, post hoc pairwise comparisions indicated its statistical equivalence with the alphabetic set. Note that averaging ranks over reviews is appropriate when reviews are treated as sampling units. Nevertheless, more nuanced analyses for review and feature type combinations revealed considerable variability. Rather than prematurely selecting the alphanumeric^+^ set as ‘best,’ a study is in progress that involves joining feature sets per review, de-duplicating, and testing. It is possible that the most informative set could be a heterogeneous mix of types and, if so, would build on results reported in studies of features in the context of work prioritization by Cohen [14] and identification of clinically rigorous research by Kilicoglu et al. [53].

Taking another tack, we tried to evaluate the relative contribution of feature types by using their prediction probabilities in a kernel logistic regression model [54]. The results were very disappointing. Although precision was good, recall was quite variable and overall performance for three of five datasets (influenza, organ transplantation, and malaria) did not surpass the baseline. We believe this is because the feature sets overlap quite a bit.

Regarding topic modeling in biomedicine, this method has been employed by mainly computational biology and bioinformatics investigators. For example, a search on 15 July 2013 of Embase, which includes MEDLINE records, indicated that *Neurocomputing* and *BMC Bioinformatics* accounted for 42% of just 38 hits. However, researchers are beginning to explore its usefulness in other areas, such as query expansion for document retrieval [55] and feature selection for automated indexing [56]. To our knowledge, using topics and KL divergences as features to classify studies for systematic reviews was novel. The strategy we used was semi-supervised in that we first fit a topic model to generate features ignoring the labels (unsupervised) and then computed KL divergences given the labels (supervised). Compared to other feature engineering efforts, fitting topic models and creating features was relatively slow. Although we have not ruled out using such features in future work, it may be that this method is more suitable for unsupervised discovery in text—its initial purpose.

Regarding extraction of SR concepts as features, our strategy was also novel for this task. However, closely related efforts were carried out by Dalal et al [13] who mapped query terms from two updated SRs to citations and by Frunza et al [18] who used protocol questions from a single review to build multiple classifiers.

The surprising similarity of performance associated with concepts in a handful of SRs written by a few reviewers as compared to terms assigned by indexers in thousands of citations was at first puzzling. On reflection, the similarity may be explained by the role of expert searchers, such as librarians and informationists. Specifically, expert searchers translate the information needs of reviewers expressed in protocols that later appear in published SRs. To do this, they develop queries using terms in the controlled vocabularies of electronic databases, including MEDLINE and Embase. These terms then show up in the indexing of the retrieved citations. Thus, the link between concepts in SRs and indexing demonstrates the apparent value of librarians and informationists who support systematic reviewers. Moreover, it probably justifies federal funding for inclusion of informationists in comparative effectiveness research [57].

### Limitations and future work

#### Simulation

Randomly sorting consensus judgments into two sets of citations was a strength of our simulation for reasons described in the Methods section. However, it was also a limitation. In reality, users would submit their judgments to a decision support system for feedback regarding their decisions before reaching consensus. The implication is that the estimated RSB based on our simulation may be optimistic. Still, reviewers do use a priori selection criteria and they usually run through calibration exercises before screening in earnest, which suggests decisions across reviewers may be reasonably coherent. If not coherent, decision support could be perceived as particularly valuable. For example, discordant decisions could be more common when a team consists of expert and novice reviewers, or methodologists and substantive domain experts.

#### Domain, complexity, and updates

We had expected that classification performance might vary with the type of systematic review. This appears to be true, although performance varies with domain and complexity rather than topic per se. For example, our test results over all conditions tended to be better for diagnostic reviews (malaria [22] and detection of galactomannan for invasive aspergillosis [23]) and worse for complicated reviews (influenza [26] and organ transplantation [25]). For example, classification of the malaria and galactomannan citations returned the best overall performance (F3) for each feature set, whereas classification of the influenza and organ transplantation citations returned the worst performance. This pattern prevails over all performance measures, with few exceptions, and may be because the diagnostic reviews addressed a single research question, whereas the complex reviews addressed three to five questions, including one question on adverse effects. The latter point is important because reports of adverse effects often appear in publication types such as *comments* or *case studies* that would otherwise render them ineligible for inclusion. Classifying such reports will become increasingly important as the Cochrane Collaboration recently issued a new mandatory standard stating that potential adverse effects must be addressed in SRs [58].

Additionally, when a review is an update, as the influenza review is [26], much can change, including the team, the searches, and even aspects of the research question [13]. Moreover, if the interim is long enough, concept drift may be a problem [59], [60]. To test the hypothesis that change affects classification, we selected citations screened in the original influenza review and extracted alphanumeric^+^ features. Note that an updated review incorporates new evidence into an earlier version and reviewers make inferences over the pooled citations [7]. As expected, performance for the original review was better than for the updated (pooled) review. Specifically, mean precision improved by 48% (from 30.08% to 44.45%) and mean classification error rate by 28% (from 12.31% to 8.88%). This suggests that a decision support system should model judgments from review versions separately if selection criteria have changed. Moreover, change across updates has implications for prioritization research (e.g., see [13]), especially since Cochrane review teams are expected to update every two years [7].

#### Misclassification

We analyzed 24 extreme cases of misclassification for five review and feature type pairs where the prediction probabilities derived from Bayesian confidences were very high. This was quite illuminating. For example, all of the selected false negatives (14/14) were in fact accurate when compared with final reviewer decisions recorded in published tables. In other words, reviewers included these studies when screening citations, but later excluded them after reading full texts. Usually, the published reasons had to do with clearly specified exclusion criteria. In this case, we would not want to change the computer's prediction, even though recall would improve, because feedback could obviate the need to read full text and therefore could save reviewers labor. On the other hand, 42% (10/24) of the misclassified cases were missing abstracts. To redress the apparent effect of paucity of information, we will create proxy abstracts in a new study by populating empty fields with text from segments of corresponding primary articles. Note that the percentage of empty abstracts ranged from 4% (ameloblastoma review) to 24% (organ transplant review). As for false positives, we should be able to devise rules or regular expressions to accurately exclude studies when the interventions or outcomes of interest are missing. Finally, we will explore using Boolean strategies that librarians use [61] in post-classifier filters to exclude studies with ineligible designs.

## Conclusions

Our results point to a promising computer-assisted decision support system for systematic review teams and solo reviewers. It seems likely that a future system based on our methods could substantially reduce the burden of screening. Additionally, such a system could deliver quality assurance both by confirming concordant decisions and by naming studies associated with discordant decisions for further consideration.

## Supporting Information

Results S1 **Review by Feature Set Results.** A more detailed presentation of the performance results for B|A and A|B independent tests, as well as mean performance. Results are presented for each systematic review by feature set. This table includes baseline F3 values, best parameter sets, recall, precision, F3, classification error, reduction in screening burden, and number of misclassified citations.
(XLSX)
